# Species identification of Middle Eastern blowflies (Diptera: Calliphoridae) of forensic importance

**DOI:** 10.1007/s00436-015-4329-y

**Published:** 2015-02-15

**Authors:** Kamran Akbarzadeh, James F. Wallman, Hana Sulakova, Krzysztof Szpila

**Affiliations:** 1 Department of Medical Entomology and Vector Control, School of Public Health, Tehran University of Medical Sciences, P.O. Box 14155/6446, Tehran, Iran; 2 School of Biological Sciences, University of Wollongong, Wollongong, NSW 2522 Australia; 3 Institute of Criminalistics Prague, P.O. Box 62/KUP, Strojnicka 27, CZ-170 89 Prague 7, Czech Republic; 4 Chair of Ecology and Biogeography, Faculty of Biology and Environmental Protection, Nicolaus Copernicus University, Lwowska 1, 87-100 Toruń, Poland

**Keywords:** Calliphoridae, Species identification, Middle East, Forensic entomology

## Abstract

The lack of reliable tools for species identification of necrophagous blowflies of the Middle East is a serious obstacle to the development of forensic entomology in the majority of countries of this region. Adding to the complexity of diagnosing the regional fauna is that species representing three different zoogeographical elements exist in sympatry. In response to this situation, a high-quality key to the adults of all species of forensically relevant blowflies of the Middle East has been prepared. Thanks to the modern technique of image-stack stereomicroscopy and high-quality entomological materials, this new key can be easily applied by investigators inexperienced in the taxonomy of blowflies. The major technical problems relating to the species identification of necrophagous blowflies of the Middle East are also discussed.

## Introduction

Necrophagous blowflies are among the most ubiquitous insects occurring in anthropogenic ecosystems. They have great medical importance, which relates to their participation in carrion decomposition, facultative parasitism of vertebrate tissues, and mechanical transmission of various pathogenic microorganisms (Norris 1965; Greenberg 1973; Hall and Wall 1995). In recent decades, there has been growing interest in research into the biology and ecology of insects with potential forensic applications (Tomberlin et al. 2011). Necrophagous blowflies, the earliest visitors to carrion as adults and the main decomposers of carrion mass as larvae, play a special role in both casework and research dedicated to medico-legal applications (Byrd and Castner 2009). The reliability of forensic entomological expertise and associated experiments strongly depends on proper species identification of collected material.

The blowflies (family Calliphoridae) in their traditional, broad form are a paraphyletic taxon. This was postulated by Rognes (1997) and has been confirmed by recent molecular studies (e.g., Kutty et al. 2010; Marinho et al. 2012; Nelson et al. 2012; Singh and Wells 2013). All species of forensically important blowflies are grouped in four subfamilies in the sense of the systematics proposed by Rognes (1991, 1997). Three of them, the Calliphorinae, Chrysomyinae, and Luciliinae, are represented in all zoogeographic regions, whereas the distribution of the Toxotarsinae is restricted to the Neotropical Region (Rognes 1997). The fauna of necrophagous blowflies has been intensively studied in Australasia, Europe, East and South Asia, and North and South America, with notable effect in the form of a variety of published checklists, keys, and taxonomic monographs (e.g., Kano and Shinonaga 1968; Kurahashi 1987; Kurahashi et al. 1997; Rognes 1991; Fan et al. 1997; Wallman 2001; Whitworth 2006; Irish et al. (2014); Yang et al. 2014). However, the blowfly fauna of some large geographical regions, like the Afrotropics (except Namibia and the Republic of South Africa) and the Middle East (except Israel), remains poorly studied. This situation stops broad application of insects for medico-legal purposes due to a lack of proper tools for species identification of the local fauna. In the Middle East, the reliable diagnosis of species is especially complicated because the constituent countries of the region lie at the intersection of three zoogeographical zones. Most Middle Eastern countries possess a blowfly fauna simultaneously representative of the Palaearctic, Oriental, and Afrotropical regions (Büttiker et al. 1979; Deeming 1996, 2007; Kurahashi and Afzal 2002; Rognes 2002). Additionally, the blowfly faunas of Bahrain, Egypt, Iran, Iraq, Jordan, Kuwait, Lebanon, Qatar, Syria, and Yemen have been studied in only a fragmentary way or not at all.

The aim of the present paper is to deliver a high-quality key for identification of the adults of all species of forensically relevant blowflies known from the Middle East. The main intention of the authors was to prepare a species identification tool that will be easily applicable to investigators inexperienced in blowfly taxonomy. This task was achieved thanks to the application of the modern technique of image-stack stereomicroscopy and the use of well-preserved entomological material. The authors hope that this publication will be a further milestone toward advancing the application of forensic entomology in Middle Eastern countries.

## Material and methods

The Middle East is defined as the group of the following countries situated in south-east Asia and northern Africa: Bahrain, Egypt, Iran, Iraq, Israel, Jordan, Kuwait, Lebanon, Libya, Qatar, Oman, Pakistan, Saudi Arabia, Syria, United Arab Emirates, Yemen, and Turkey (Kort 2008). The relevant species of blowflies of forensic importance known from the Middle East, and their distributions, were determined using the catalogs of Schumann (1986) and Verves (2005), with the addition of the following publications: Büttiker et al. (1979), Deeming (1996, 2007), Parchami-Araghi et al. (2001), Kurahashi and Afzal (2002), Rognes (2002), Al-Mesbah (2010), Sabanoğlu and Sert (2010), Tüzün et al. (2010), Abouzied (2014) and Verves and Khrokalo (2014). However many records reported in the listed references need verification and should be treated with caution. New distribution records for some species for specific countries resulted from the inspection of regional collections by the authors. These new records are noted in the key by underlining the names of the countries concerned.

Most of the specimens used for the illustration of characters were collected personally by the authors. Specimens of other species, especially nominal elements of the Oriental and Afrotropical fauna, were obtained thanks to loans from the collection of the Natural History Museum of Denmark.

The preparation of image-stacking was done using an M205C Leica Stereomicroscope with an integrated high-resolution Leica DFC495 digital camera and associated software (Leica Application Suite 4.4.0).

Characters used in the key are mostly compiled from the following sources: Rognes (1991), Kurahashi et al. (1997), Fan et al. (1997), Wallman (2001), Rognes and Paterson (2005), Szpila (2012), Grella et al. (2015), and Williams and Villet (2014).

The terminology follows Rognes (1991), and all morphological details are clearly marked and abbreviated on the figures.

## Results

Key to the identification of forensically important blowflies of the Middle East.

1. Stem-vein without row of hairs above … 2
  stem-vein with row of hairs above (Fig. 1a) … 3 (Chrysomyinae)

**Fig. 1:**
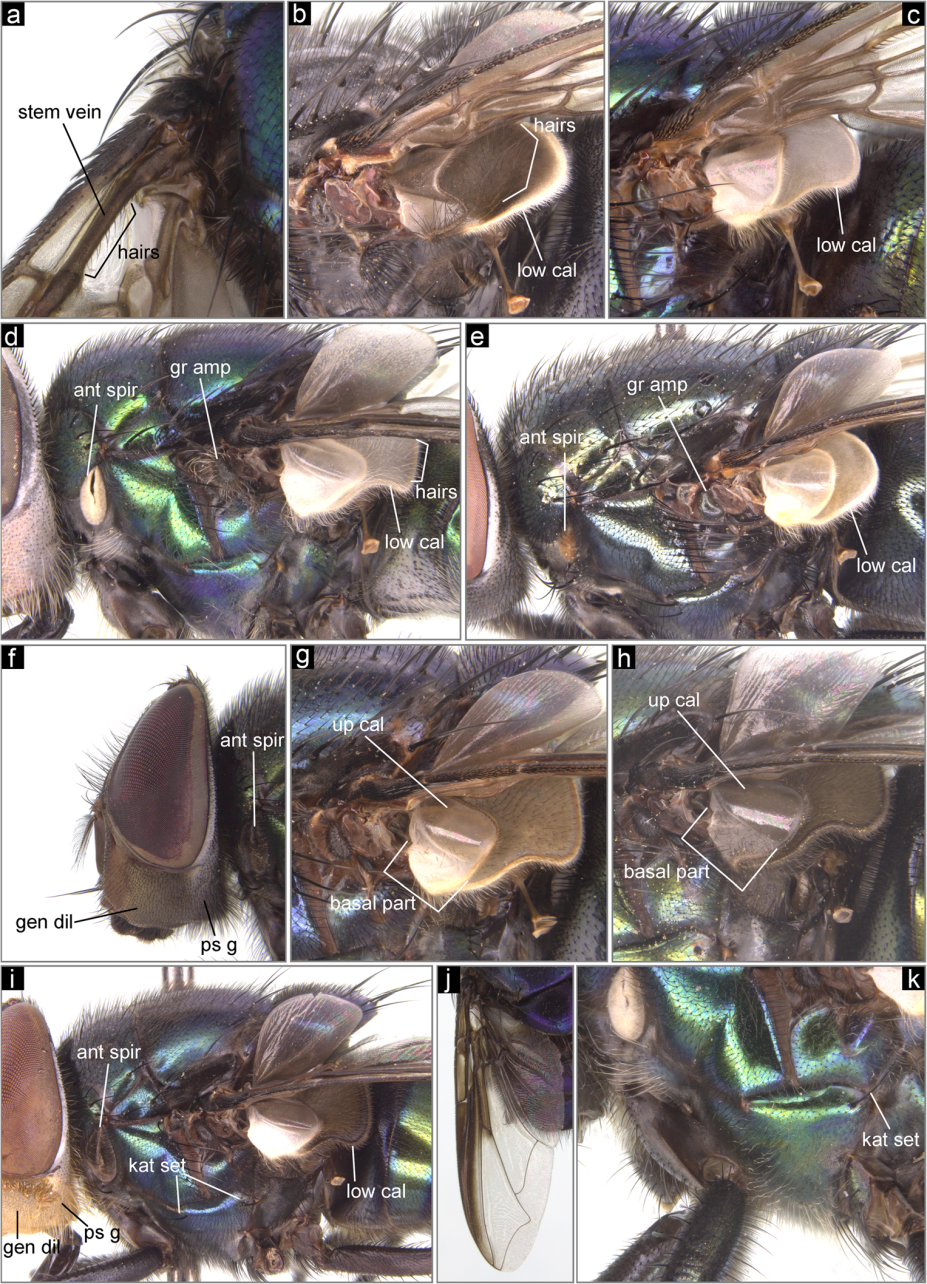
The Middle Eastern blowflies of forensic importance. **a** *Ch. albiceps*, basal part of wing, stem vein. **b** *C. vicina*, thorax, upper, and lower calypters. **c** *L. caesar*, thorax, upper, and lower calypters. **d** *Ch. albiceps*, thorax, lateral view. **e** *P. regina*, thorax, lateral view. **f** *Ch. pinguis*, head, lateral view. **g** *Ch. phaonis*, thorax, upper, and lower calypters. **h** *Ch. pinguis*, thorax, upper, and lower calypters. **i** *Ch. megacephala*, thorax, lateral. **j** *Ch. marginalis*, wing. **k** *Ch. nigripes*, thorax, lateral view. *ant spir* anterior spiracle, *gen dil* genal dilation, *gr amp* great ampulla, *kat set* katepisternal setae, *low cal* lower calypter, *ps g* postgena, *up cal* upper calypter
2. Lower calypter with hairs on dorsal surface; thorax nonmetallic, dark and dusted (Fig. 1b) … 13 (Calliphorinae)
  lower calypter without hairs on dorsal surface; thorax bright green metallic, rarely bluish or cuprous (Fig. 1c) … 15 (Luciliinae)
3. Greater ampulla with stiff erect hairs (Fig. 1d); dorsal surface of lower calypter with dense hairs (Fig. 1d) … 4 (*Chrysomya* spp.)
  greater ampulla bare or with short fine hairs (Fig. 1e); dorsal surface of lower calypter bare or with a few pale hairs (Fig. 1e) … 12
4. Anterior spiracle dark, brownish (Fig. 1f, i) … 5
  anterior spiracle bright, white-yellowish (Figs 1d, k; 2g) … 8

**Fig. 2:**
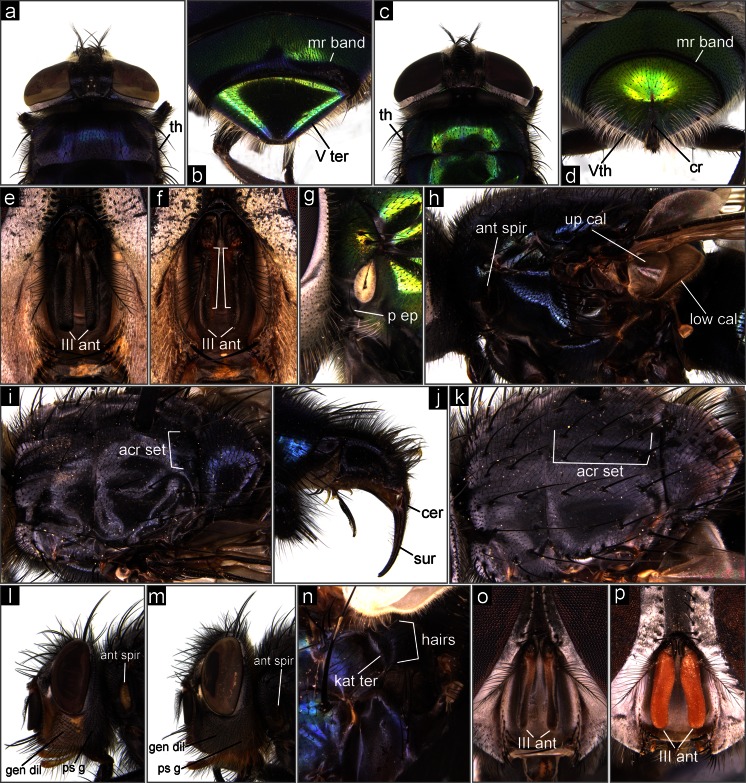
The Middle Eastern blowflies of forensic importance. **a** *Ch. putoria*, anterior part of body, dorsal view. **b** *Ch. putoria*, distal part of female abdomen, postero-dorsal view. **c** *Ch. albiceps*, anterior part of body, dorsal view. **d** *Ch. albiceps*, distal part of female abdomen, postero-dorsal view. **e** *Ch. albiceps*, head, antennae. **f** *Ch. rufifacies*, head, antennae. **g** *Ch. rufifacies*, anterior part of thorax, lateral view. **h** *P. terraenovae*, thorax, lateral view. **i** *C. mortuorum*, thorax, dorsal view. **j** *C. mortuorum*, posterior part of abdomen, male genital apparatus. **k** *C. vicina*, thorax, dorsal view. **l** *C. vicina*, head, lateral view. **m** *C. vomitoria*, head, lateral view. **n** *H. pulchra*, posterior part of thorax, lateral view. **o** *H. ligurriens*, head, antennae. **p** *H. pulchra*, head, antennae. *acr set* acrostichal setae, *ant spir* anterior spiracle, *cer* cercus, *cr* crevice/incision, *gen dil* genal dilation, *III ant* third antennal segment, *kat ter* katatergite, *low cal* lower calypter, *mr band* marginal band, *p ep* proepimeral seta, *ps g* postgena, *sur* surstylus, *th* thorax, *up* upper calypter, *V ter* / *Vth* fifth tergite
5. Surface of genal dilation and postgena fuscous, with black hairs (Fig. 1f) … 6
  surface of genal dilation and postgena bright, orange-yellowish, with yellow hairs (Fig. 1i) … 7
6. Basal part of upper calypter clearly bright, almost white; remaining part of upper calypter and lower calypter dirty-white (Fig. 1g) … *Chrysomya phaonis* Séguy, 1928
  Distribution: Pakistan. Possible occurrence in south-east Iran.
  basal part of upper calypter dark gray; remaining part of upper calypter and lower calypter dark brownish (Fig. 1h) … *Chrysomya pinguis* (Walker, 1858)
  Distribution: Pakistan. Possible occurrence in south-east Iran.
7. Lower calypter white, with yellowish fringe (Fig. 1d) … *Chrysomya bezziana* Villeneuve, 1914
  Distribution: Bahrain, Iran, Iraq, Kuwait, Oman, Pakistan, Qatar, Saudi Arabia, United Arab Emirates. Possible occurrence in all countries of the Middle East.
  lower calypter brownish and darkened (Fig. 1i) … *Chrysomya megacephala* (Fabricius, 1794)
  Distribution: Egypt, Iran, Kuwait, Oman, Pakistan, Saudi Arabia, United Arab Emirates. Possible occurrence in all countries of the Middle East.
8. Anterior wing margin strongly darkened (Fig. 1j) … *Chrysomya marginalis* (Wiedemann, 1830)
  Distribution: Egypt, Iran, Israel, Oman, Pakistan, Saudi Arabia, Syria, United Arab Emirates. Possible occurrence in all countries of the Middle East.
  anterior wing margin transparent … 9
9. Katepisternal setae 0 + 1 (Fig. 1k); all hairs on surface of tergite V black … *Chrysomya nigripes* Aubertin, 1932
  Distribution: Pakistan. Possible occurrence in south-east Iran.
  katepisternal setae 1 + 1 (Fig. 1i); at least some hairs on lateral surfaces of tergite V white (Fig. 2d) … 10
10. Dorsal part of thorax with conspicuous dusting (Fig. 2a); black transverse marginal abdominal bands on abdominal segment III broad, even up to one-half of tergite length (Fig. 2b); posterior edge of tergite V of female entire, without incision (Fig. 2b) … *Chrysomya putoria* (Wiedemann, 1830)
  Distribution: Saudi Arabia (?). Records of Büttiker et al. (1979) and Abouzied (2014) of "*Chrysomya chloropyga*" most likely refer to *Ch. putoria*. Possible occurrence in countries of Arabic Peninsula. Reliable keys for identification of both species are provided by Rognes and Paterson (2005) and Irish et al. (2014).
  dorsal part of thorax shiny, with little dusting (Fig. 2c); black transverse marginal abdominal bands on abdominal segments III and IV very narrow, up to about a quarter on segment III and usually not more than about one sixth in segment IV (Fig. 2d); posterior edge of tergite V of female with crevice/incision (Fig. 2d) … 11
11. Third antennal segment wholly dark, blackish-brownish (Fig. 2e); proepimeral seta absent (Fig. 1d) (rarely present on one or both sides) … *Chrysomya albiceps* (Wiedemann, 1819)
  Distribution: Egypt, Israel, Iran, Iraq, Kuwait, Lebanon, Libya, Oman, Pakistan, Saudi Arabia, Syria, United Arab Emirates, Turkey. Possible occurrence in all countries of the Middle East.
  third antennal segment pale brown-reddish on inner surface (Fig. 2f); proepimeral seta present (Fig. 2g) … *Chrysomya rufifacies* (Macquart, 1843)
  Distribution: Iran, Pakistan.
12. Upper and lower calypters bright, white to yellowish (Fig. 1e); anterior spiracle yellowish (Fig. 1e) … *Phormia regina* (Meigen, 1826)
  Distribution: Pakistan. Possible occurrence in Turkey and north-west Iran.
  upper and lower calypters dark brown, especially on rim (Fig. 2h); anterior spiracle dark brown (Fig. 2h) … *Protophormia terraenovae* (Robineau-Desvoidy, 1830)
  Distribution: Pakistan. Possible occurrence in Turkey and north-west Iran.
13. One pair of acrostichal setae on postsutural surface of thorax (Fig. 2i); abdomen shining blue without dusting; cerci of male genitalia short, surstyli much larger in form of long slightly curved rods (Fig. 2j) … *Cynomya mortuorum* (Linnaeus, 1761)
  Distribution: Pakistan. Possible occurrence in Turkey and north-west Iran.
  Three pairs of acrostichal setae on postsutural surface of thorax (Fig. 2k); abdomen shining blue with white dusting; cerci and surstyli almost the same length … 14 (*Calliphora* spp.)
14. facial ridges, mouth edge, and anterior part of genal dilation yellowish-red (Fig. 2l); hairs on genal dilation and postgena black (Fig. 2l); anterior spiracle yellow (Fig. 2l) … *Calliphora vicina* (Robineau-Desvoidy, 1830)
  Distribution: Egypt, Iran, Iraq, Israel, Jordan, Kuwait, Lebanon, Pakistan, Saudi Arabia, Syria, Turkey, Yemen. Possible occurrence in all countries of the Middle East.
  facial ridges, mouth edge, and anterior part of genal dilation black (Fig. 2m); hairs on posterior part of genal dilation and postgena orange (Fig. 2m); anterior spiracle brownish-black (Fig. 2m) … *Calliphora vomitoria* (Linnaeus, 1758)
  Distribution: Iran, Israel, Pakistan, Saudi Arabia, Syria, Turkey.
15. Katatergite bare or pubescent (Fig. 3h, i) … 16 (*Lucilia* spp.)

**Fig. 3:**
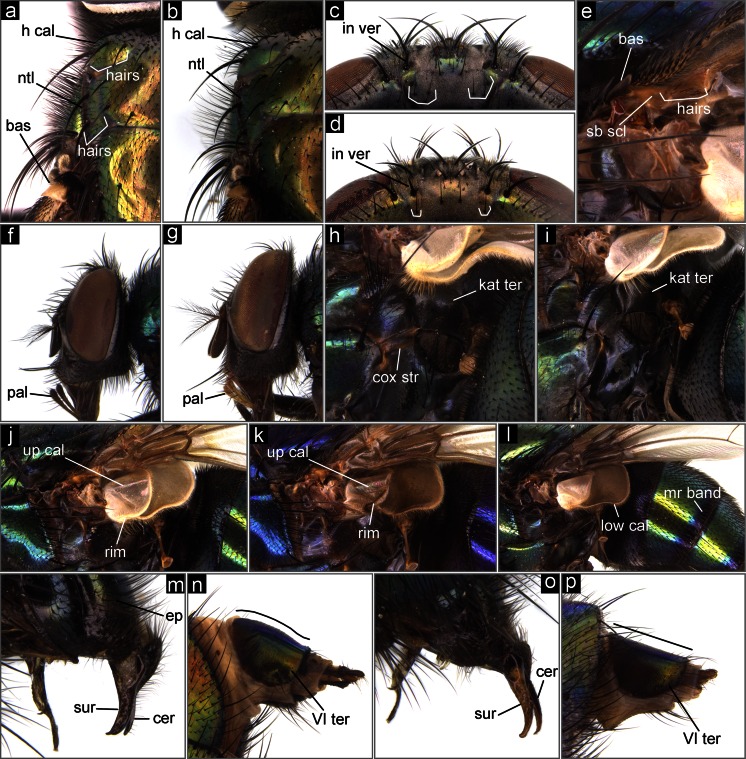
The Middle Eastern blowflies of forensic importance. **a** *L. sericata*, thorax, dorsal view. **b** *L. cuprina*, thorax, dorsal view. **c** *L. sericata*, head, posterior view. **d** *L. cuprina*, head, posterior view. **e** *L. illustris*, base of wing, ventral view. **f** *L. silvarum*, head, lateral view. **g** *L. ampullacea*, head, lateral view. **h** *L. illustris*, posterior part of thorax, lateral view. **i** *L. ampullacea*, posterior part of thorax, lateral view. **j** *L. ampullacea*, upper and lower calypters, lateral view. **k** *L. porphyrina*, upper, and lower calypters, lateral view. **l** *L. papuensis*, upper and lower calypters, lateral view. **m** *L. caesar*, posterior part of abdomen, male genital apparatus. **n** *L. caesar*, ovipositor, VIth tergite, lateral view. **o** *L. illustris*, posterior part of abdomen, male genital apparatus. **p** *L. illustris*, ovipositor, VIth tergite, lateral view. *bas* basicosta, *cer* cercus, *cox str* coxopleural streak, *ep* epandrium, *h cal* humeral callus, *in ver* inner vertical seta, *kat ter* katatergite, *low cal* lower calypter, *mr band* marginal band, *ntl* notopleuron, *pal* palpus, *sb scl* subcostal sclerite, *sur* surstylus, *up cal* upper calypter, *VI ter* sixth tergite

  katatergite with long, upstanding fine hairs (Fig. 2n) … 23 (*Hemipyrellia* spp.)
16. Basicosta bright yellow (Fig. 3a) … 17
  basicosta brown or black (Fig. 3e) … 18
17. Posterior slope of humeral callus with 6–8 hairs (Fig. 3a); surface of notopleuron between last notopleural seta and edge of notopleuron with 8–16 hairs (Fig. 3a); central occipital area with 2–8 setulae (rarely 1) below each inner vertical seta (Fig. 3c) … *Lucilia sericata* (Meigen, 1826)
  Distribution: Egypt, Iran, Iraq, Israel, Jordan, Kuwait, Lebanon, Libya, Oman, Pakistan, Saudi Arabia, Syria, Turkey, Yemen. Possible occurrence in all countries of the Middle East.
  posterior slope of humeral callus with 0–4 hairs (Fig. 3b); surface of notopleuron between last notopleural seta and edge of notopleuron with 2–5 hairs (Fig. 3b); central occipital area with one setula (rarely 0 or 2) below each inner vertical seta (Fig. 3d) … *Lucilia cuprina* (Wiedemann, 1830)
    Distribution: Egypt, Iran, Iraq, Israel, Pakistan, Turkey. Possible occurrence in all countries of the Middle East.
18. Three pairs of acrostichal setae on postsutural area of thorax (like on Fig. 2k); ventral surface of subcostal sclerite without black setulae; palpi brown to black (Fig. 3f) … *Lucilia silvarum* (Meigen, 1826)
  Distribution: Iran, Israel.
  Two pairs of acrostichal setae on postsutural area of thorax; ventral surface of subcostal sclerite with black setulae near apex (Fig. 3e); palpi yellow-orange (Fig. 3g) … 19
19. Coxopleural streak absent (Fig. 3i) … 20
  coxopleural streak present (Fig. 3h) … 21
20. Calypters white to light brown, with white at least along the rim of upper calypter (Fig. 3j); tibia of legs black; body usually green … *Lucilia ampullacea* Villeneuve, 1922
  Distribution: Pakistan. Possible occurrence in Turkey and north-west Iran.
  calypters light brown to brown, with at least the rim of upper calypter brownish (Fig. 3k); tibia of legs orange-brownish; body usually bluish-purple … *Lucilia porphyrina* (Walker, 1856)
  Distribution: Pakistan. Possible occurrence in south-east Iran.
21. At least lower calypter brown (Fig. 3l); abdominal segments III and IV with dark marginal band (resembling bands present in *Chrysomya* spp.) (Fig. 3l) … *Lucilia papuensis* Macquart, 1843
  Distribution: Pakistan. Possible occurrence in south-east Iran.
  lower calypter white, sometimes slightly darkened but never brown (Fig. 1c); abdominal segments III and IV with without dark marginal band … 22
22. Male: epandrium large and swollen (Fig. 3m); surstyli stout, abruptly narrowed at tip (Fig. 3m); female: tergite VI convex in lateral view (Fig. 3n) … *Lucilia caesar* (Linnaeus, 1758)
  Distribution: Egypt, Iran, Iraq, Israel, Jordan, Lebanon, Libya, Oman, Saudi Arabia, Syria, Turkey, Yemen. Possible occurrence in mountain zone of Pakistan.
  male: epandrium of normal size (Fig. 3o); surstyli slender, gradually tapered to sharp tip (Fig. 3o); female: tergite VI straight in lateral view (Fig. 3p) … *Lucilia illustris* (Meigen, 1826)
  Distribution: Egypt, Iran, Iraq, Israel, Jordan, Saudi Arabia, Syria, Turkey, Yemen.
23. Third antennal segment dark, slightly orange at the base (Fig. 2o) … *Hemipyrellia ligurriens* (Wiedemann, 1830)
  Distribution: Pakistan. Possible occurrence in south-east Iran.
  third antennal segment entirely orange (Fig. 2p) … *Hemipyrellia pulchra* (Wiedemann, 1830)
  Distribution: Egypt, Pakistan. Possible occurrence in southern Middle East.

## Discussion

The present key covers 24 species of necrophagous blowflies. The undoubted forensic importance of most of them has been discussed and summarized in monographs (e.g., Byrd and Castner 2009; Gennard 2012; Rivers and Dahlem 2014) and some recent papers (Sukontason et al. 2010; Szpila 2010; Kurahashi and Kirk-Spriggs 2006; Klong-Klaew et al. 2012). The list of flies of forensic importance is not finite; new species are consequently being added based on new information (e.g., Vanin et al. 2011; Fremdt et al. 2012; Grzywacz et al. 2014). Therefore, necrophagous species like *Ch. pinguis*, *Ch. phaonis*, *L. papuensis*, *H. pulchra*, abundant in anthropogenic habitats but still without confirmed attraction to human corpses, have also been included in the key presented here. In the high mountain zones of Pakistan and Iran, it is possible to find additional species of *Calliphora* Robineau-Desvoidy, 1830 (Schumann 1986; Kurahashi and Afzal 2002): *Calliphora chinghaiensis* Van et Ma, 1978, *Calliphora himalayana* Kurahashi, 1994, and *Calliphora uralensis* Villeneuve, 1922. However, either their biology remains unknown or they do not show propensity for development in large vertebrate carrion. They have therefore not been included in the present key.

Species identification of the adults of necrophagous blowflies is usually not difficult, and numerous keys are currently available for potential users (e.g., Fan et al. 1997; Kano and Shinonaga 1968; Kurahashi 1987; Kurahashi et al. 1997; Rognes 1991; Wallman 2001; Whitworth 2006; Nazni et al. 2011; Szpila 2012). Given this situation, many tested morphological characters have been available for the preparation of a reliable key for the identification of material collected in Middle Eastern countries. However, some characters must nonetheless be treated with caution:

1. Color and dusting of particular elements of the body. This character may be difficult to observe if specimens are excessively weathered, or when they have been preserved in EtOH or stored too long in an ethyl acetate killing jar. Restoration of the colors and dusting of material preserved in EtOH can usually be achieved by brief immersion in distilled water, followed by about 5 min of exposure to the heat from the incandescent bulb of a simple table lamp. Longer drying may cause undesirable shrinkage and wrinkling of specimens. Specimens damaged by contact with ethyl acetate can be restored by placing them in a bath of xylene for 24 h.
2. Presence of setae. Strong setae on the bodies of flies are prone to damage, again, especially in specimens that are weathered or that have been preserved in EtOH. However, even if setae are broken off, their presence or absence may be checked by the presence of the clear round socket which always remains visible after removal of a seta. It is important to mention that populations of flies always contain a proportion of specimens with aberrations in size and in the position of particular setae (Rognes 1991). Therefore, to minimize such potential ambiguity, characters in the present key concerned with setulation are always supplemented by other features.
3. Genital apparatus. Features of the genitalia are always the most reliable characters for species identification. The male genital structures are easily observed in specimens preserved in 70 % EtOH. However, specimens preserved in absolute EtOH or pinned (if the genital apparatus was not exposed before drying out) need special treatment. The procedure for such dry specimens was described in detail by Rognes (1991). Rigid (dehydrated) specimens after preservation in absolute EtOH may be softened by boiling for 5 min in a 5 % solution of KOH. An alternative is the relocation and storage of specimens to a more dilute EtOH solution. The disadvantage of the latter method is that it takes longer to achieve the softening effect.

The Middle East is a region in which three pairs of sister species occur sympatrically: *L. cuprina* and *L. sericata*, *L. caesar* and *L. illustris* and *Ch. albiceps* and *Ch. rufifacies*. The morphological separation of species of *Lucilia* is not especially controversial thanks to former and more recent detailed studies of these species (Rognes 1991, 1994; Williams and Villet 2014). Details of the male genital apparatus, especially the shape of the cerci and surstyli, are reliable characters for differentiation of particular species, supported by quite a large set of other characters in the case of *L. cuprina* and *L. sericata* (Williams and Villet 2014). Identification of the females of *L. caesar* and *L. illustris* is possible by checking the shape of the dorsal surface of the sixth tergite and the distribution and size of setae on the posterior margin of the same tergite (Rognes 1991).

The identification and overall taxonomy of *Ch. albiceps* and *Ch. rufifacies* are problematic and have been discussed by several past workers (e.g., Holdaway 1933; Zumpt 1956; Guimarães et al. 1978; Tantawi and Greenberg 1993; Whitworth 2006). The few available characters are not fully reliable, such as the presence of the proepimeral seta (Tantawi and Greenberg 1993), or are difficult to observe and prone to damage, like the color of inner surface of third antennal segment (Holdaway 1933). The results of intensive studies of the problem have only just been published, but the characters proposed for the differentiation of specimens of both species are still very subtle (Grella et al. 2015). For example, the color of the genal dilation and the angle formed by the branch of vein M1 + 2 are difficult for inexperienced researchers to interpret, and the number of proepisternal setae may only be clearly visible after removal of the head of the specimen. However, in the absence of morphological certainty, the identity of *Ch. albiceps* and *Ch. rufifacies* can be corroborated by molecular analysis.

The politically fraught conditions in many of the countries of the Middle East pose obvious difficulties for the study of the blowflies and other components of the natural history of the region. Even so, when secure opportunities present themselves, we encourage workers to continue to document the blowfly species that occur in this part of the world to help facilitate the diverse applications of these important insects.

## References

[CR1] Abouzied EM (2014) Insect colonization and succession on rabbit carcasses in southwestern mountains of the Kingdom of Saudi Arabia. J Med Entomol 56:1168–1174 10.1603/ME13181 26309303

[CR2] Al-Mesbah HA (2010) A study of forensically important necrophagous Diptera in Kuwait. University of Central Lancashire, Faculty of Science, Forensic and Investigation Department, M.Sc. thesis

[CR3] Büttiker W, Attiah MD, Pont AC (1979). Insects of Saudi Arabia. Diptera: synanthropic flies. Fauna Saudi Arab.

[CR4] Byrd JH, Castner JL (2009) Insects of forensic importance. In: Byrd JH, Castner JL (eds) Forensic Entomology: The Utility of Arthropods in Legal Investigations. CRC Press, Boca Raton, II edition, pp 43–79

[CR5] Deeming JC (1996). The Calliphoridae (Diptera: Cyclorrhapha) of Oman. Fauna Saudi Arab.

[CR6] Deeming JC (2007). Order Diptera, family Calliphoridae. Arthropod Fauna UAE.

[CR7] Fan Z, Zhizi C, Jianming F, Shensheng Z, Zhenliang T (1997). Diptera: Calliphoridae. Fauna Sinica, Insecta, Vol. 6.

[CR8] Fremdt H, Szpila K, Huijbregts H, Lindström A, Zehner R, Amendt J (2012). *Lucilia silvarum* Meigen, 1826 (Diptera: Calliphoridae) – a new species of interest for forensic entomology in Europe. Forensic Sci Int.

[CR9] Gennard D (2012). Forensic entomology, an introduction.

[CR10] Greenberg B (1973). Flies and diseases. Biology and disease transmission, Vol. 2.

[CR11] Grella MD, Savino AG, Paulo DF, Mendes FM, Azeredo-Espin AML, Queiroz MMC, Thyssen PJ, Linhares AX (2015) Phenotypic polymorphism of *Chrysomya albiceps* (Wiedemann) (Diptera: Calliphoridae) may lead to species misidentification. Acta Trop 141:60–72 10.1016/j.actatropica.2014.09.011 25265317

[CR12] Grzywacz A, Lindström A, Hall MJR (2014). *Hydrotaea similis* Meade (Diptera: Muscidae) newly reported from a human cadaver: A case report and larval morphology. Forensic Sci Int.

[CR13] Guimarães JH, Prado AP, Linhares AX (1978). Three newly introduced blowflies species in Southern Brazil (Diptera, Calliphoridae). Rev Bras Entomol.

[CR14] Hall MJR, Wall R (1995). Myiasis of humans and domestic animals. Adv Parasitol.

[CR15] Holdaway FG (1933). The synonymy and distribution of *Chrysomya rufifacies* (Macq.), an Australian sheep blowfly. Bull Entomol Res.

[CR16] Irish S, Lindsay T, Wyatt N (2014) Key to adults of Afrotropical species of the genus *Chrysomya* Robineau-Desvoidy (Diptera: Calliphoridae). African Entomol 22:297–306

[CR17] Kano R, Shinonaga S (1968). Calliphoridae (Insecta: Diptera). Fauna Japonica.

[CR18] Klong-Klaew T, Sukontason K, Sribandtimongkol P, Moophayak K, Sanit S, Sukontason KL (2012). Observations on morphology of immature *Lucilia porphyrina* (Diptera: Calliphoridae), a fly species of forensic importance. Parasitol Res.

[CR19] Kort MG (2008). The Handbook of the Middle East.

[CR20] Kurahashi H (1987). The blow flies of the New Guinea, Bismarck Archipelago and Bougainville Island.

[CR21] Kurahashi H, Afzal M (2002). The blow flies recorded from Pakistan, with the description of one new species (Diptera: Calliphoridae). Med Entomol Zool.

[CR22] Kurahashi H, Kirk-Spriggs A (2006). The Calliphoridae of Namibia (Diptera: Oestroidea). Zootaxa.

[CR23] Kurahashi H, Benjaphong N, Omar B (1997) Blow flies (Insecta: Diptera: Calliphoridae) of Malaysia and Singapore. School of Biological Sciences, University of Singapore

[CR24] Kutty SN, Pape T, Wiegmann BM, Meier R (2010). Molecular phylogeny of the Calyptratae (Diptera: Cyclorrhapha) with an emphasis on the superfamily Oestroidea and the position of Mystacinobiidae and McAlpine's Fly. Syst Entomol.

[CR25] Marinho MAT, Junqueira ACM, Paulo DF, Esposito MC, Villet MH, Azaredo-Espin AML (2012). Molecular phylogenetics of Oestroidea (Diptera; Calyptratae) with emphasis on Calliphoridae: Insights into the inter-familiar relationships and additional evidence for paraphyly among blowflies. Mol Phylogenet Evol.

[CR26] Nazni WA, Jeffery J, Heo CC, Chew WK, Lee HL (2011). Illustrated keys to adult flies of forensic importance in Malaysia.

[CR27] Nelson LA, Lambkin CL, Batterham P, Wallman JF, Dowton M, Whiting MF, Yeates DK, Cameron SL (2012). Beyond barcoding: A mitochondrial genomics approach to molecular phylogenetics and diagnostic of blowflies (Diptera: Calliphoridae). Gene.

[CR28] Norris KR (1965) The bionomics of blowflies. Annu Rev Entomol 10:47–68

[CR29] Parchami-Araghi M, Peris SV, Gonzáles-Mora D (2001). New records of Iranian Calliphoridae and Sarcophagidae, with a guide to the males of Palaearctic *Protocalliphora* (Diptera, Calyptratae). Bol R Soc Esp Hist Nat (Sec Biol).

[CR30] Rivers DB, Dahlem GA (2014) The science of forensic entomology. Wiley & Blackwell, Chichester

[CR31] Rognes K (1991). Blowflies (Diptera, Calliphoridae) of Fennoscandia and Denmark. Fauna Entomologica Scandinavica, Vol. 24.

[CR32] Rognes K (1994). First record of the sheep greenbottle fly *Lucilia cuprina* (Wiedemann, 1830) from Europe (Diptera: Calliphoridae) with additional Spanish records of Calliphoridae, Muscidae and Sarcophagidae. Eos.

[CR33] Rognes K (1997). The Calliphoridae (blowflies) (Diptera: Oestroidea) are not a monophyletic group. Cladistics.

[CR34] Rognes K (2002) Blowflies (Diptera: Calliphoridae) of Israel and adjacent areas, including a new species from Tunisia. Entomologica Scandinavica suppl. No. 59

[CR35] Rognes K, Paterson HEH (2005). *Chrysomya chloropyga* (Wiedemann, 1818) and *Chrysomya putoria* (Wiedemann, 1830) are two different species. Afr Entomol.

[CR36] Sabanoğlu B, Sert S (2010). Determination of Calliphoridae (Diptera) fauna and seasonal distribution on carrion in Ankara Province. J Forensic Sci.

[CR37] Schumann H (1986) The Calliphoridae. In: Soos A, Papp L (eds) Catalogue of Palaearctic Diptera 12, p 11

[CR38] Singh B, Wells JD (2013). Molecular systematics of the Calliphoridae (Diptera: Oestroidea): Evidence from one mitochondrial and three nuclear genes. J Med Entomol.

[CR39] Sukontason K, Sribanditmongkol P, Ngoen-klan R, Klong-klaew T, Moophayak K, Sukontason KL (2010). Differentiation between *Lucilia cuprina* and *Hemipyrellia ligurriens* (Diptera: Calliphoridae) larvae for use in forensic entomology applications. Parasitol Res.

[CR40] Szpila K, Amendt J, Goff ML, Campobasso CP, Grassberger M (2010). Key for the identification of third instars of European blowflies (Diptera: Calliphoridae) of forensic importance. Current concepts in forensic entomology.

[CR41] Szpila K (2012) Key for identification of European and Mediterranean blowflies (Diptera, Calliphoridae) of medical and veterinary importance – adult flies. In: Gennard D (ed) Forensic entomology, an introduction, II edition. Willey-Blackwell, pp. 77–81 + plates 5.1–5.9

[CR42] Tantawi TI, Greenberg B (1993). *Chrysomya albiceps* and *C. rufifacies* (Diptera: Calliphoridae): contribution to an ongoing taxonomic problem. J Med Entomol.

[CR43] Tomberlin JK, Mohr R, Benbow ME, Tarone AM, VanLaerhoven S (2011). A roadmap for bridging basic and applied research in forensic entomology. Annu Rev Entomol.

[CR44] Tüzün A, Dabiri F, Yüksel S (2010). Preliminary study and identification of insects’ species of forensic importance in Urmia, Iran. Afr J Biotechnol.

[CR45] Vanin S, Gherardi M, Bugelli V, Di Paolo M (2011). Insects found on a human cadaver in central Italy including the blowfly *Calliphora loewi* (Diptera, Calliphoridae), a new species of forensic interest. Forensic Sci Int.

[CR46] Verves YG (2005). A catalogue of Oriental Calliphoridae (Diptera). Dipterological Res.

[CR47] Verves YG, Khrokalo LA (2014) The flies (Diptera: Calliphoridae, Sarcophagidae, Rhinophoridae) of M. M. Gryshko National Botanical Garden. Ukrainskaya Entomofaunistika 5:11–28

[CR48] Wallman JF (2001) A key to the adults of species of blowflies in southern Australia known or suspected to breed in carrion [corrigendum in Med Vet Entomol 16:223]. Med Vet Entomol 15:433–437 10.1046/j.0269-283x.2001.00331.x 11776462

[CR49] Whitworth T (2006). Keys to the genera and species of blow flies (Diptera: Calliphoridae) of America north of Mexico. Proc Entomol Soc Wash.

[CR50] Williams KA, Villet MH (2014). Morphological identification of *Lucilia sericata*, *Lucilia cuprina* and their hybrids (Diptera, Calliphoridae). ZooKeys.

[CR51] Yang S-T, Kurahashi H, Shiao S-F (2014). Keys to the blow flies of Taiwan, with a checklist of recorded species and the description of a new species of *Paradichosia* Senior-White (Diptera, Calliphoridae). ZooKeys.

[CR52] Zumpt F, Lindner E (1956). 64i. Calliphorinae. Die Fliegen der Palaearktischen Region, Lieferung 190.

